# Validation of GOAL questionnaire as screening tool for clinical obstructive sleep apnea: A large sample study in China

**DOI:** 10.3389/fnins.2022.1046603

**Published:** 2022-11-04

**Authors:** Zhenzhen Zheng, Jinru Zhu, Hongwei Liang, Chaoyu Wang, Mingdi Chen, Chunhe Li, Zhiping Zhang, Riken Chen, Kang Wu, Wang Liu

**Affiliations:** ^1^Department of Respiration, The Second Affiliated Hospital of Guangdong Medical University, Zhanjiang, China; ^2^Department of Pulmonary and Critical Care Medicine, The People’s Hospital of Jiangmen, Jiangmen Hospital, Southern Medical University, Jiangmen, China; ^3^Department of Respiration, Taishan Hospital of Traditional Chinese Medicine, Jiangmen, China; ^4^Department of Critical Care Medicine, The Second Affiliated Hospital of Guangdong Medical University, Zhanjiang, China; ^5^Department of Critical Care Medicine, The First Affiliated Hospital of Guangzhou University of Chinese Medicine, Guangzhou, China; ^6^National Clinical Research Center for Respiratory Disease, State Key Laboratory of Respiratory Disease, Guangzhou Institute of Respiratory Health, The First Affiliated Hospital of Guangzhou Medical University, Guangzhou, China

**Keywords:** GOAL questionnaire, NoSAS score, STOP-Bang, ESS score, diagnosis, sleep apnea syndrome

## Abstract

**Background:**

Obstructive sleep apnea (OSA) is a serious disease with a high prevalence in the general population. The purpose of this study is to explore the effectiveness of the GOAL questionnaire in the clinical screening of OSA and compare it with other existing screening tools.

**Materials and methods:**

Outpatients and inpatients who underwent polysomnography (PSG) examination at the Sleep Medicine Center of the First Affiliated Hospital of Guangzhou Medical University from January 2013 to November 2016 were analyzed retrospectively. The basic data such as demographic, medical history, etc., and PSG data of the patients were collected, and the sensitivity, specificity, positive predictive value, negative predictive value and area under the curve (AUC) of GOAL and five other screening scales (the NoSAS score, Epworth Sleepiness Scale, the Berlin questionnaire, STOP, and STOP-Bang questionnaire) were calculated.

**Results:**

Data from 2,171 participants (1,644 male; 78%) were analyzed there were 1,507 OSA patients [Apnea Hypopnea Index (AHI) ≥ 5 events/h] among them, accounting for about 69.415%. No matter which cut-off point (AHI ≥ 5, 15 and 30 events/h), the AUC score reveals that GOAL questionnaire had comparable screening ability to the NoSAS and STOP-BANG, and performed better than the ESS, and the AUC scores of the STOP questionnaire and Epworth Sleepiness Scale (ESS) were both lower than 0.7. When the cut-off point of the AHI was 5 events/h, the AUC of GOAL was the highest at 0.799 (0.781–0.816), and its sensitivity was the highest at 89.1%. The sensitivity levels of the NoSAS score and STOP-Bang questionnaire were 67.4 and 78.8% respectively, while ESS and the Berlin questionnaire have higher specificity (70.2 and 72.3% respectively) but lower sensitivity (49.3 and 60.0% respectively).

**Conclusion:**

GOAL is a free, efficient and easy to manage tool with a screening ability comparable to NoSAS and STOP-Bang, and better than that of ESS.

## Introduction

Obstructive sleep apnea (OSA) is a common frequently-occurring disease. Due to obesity, changes in the muscle function of the upper respiratory tract, pharyngeal neuropathy and other factors, the throat will repeatedly narrow or collapse during sleep, resulting in intermittent hypoxia and carbon dioxide increase, these conditions will occur repeatedly and increased breathing during sleep (Lévy et al., 2015). A growing number of studies have shown that OSA can increase the occurrence and development of coronary heart disease, heart failure, stroke and atrial fibrillation (Drager et al., 2017); increase the risk of cognitive impairment, dementia, and Alzheimer’s disease (AD) (Leng et al., 2017; Bubu et al., 2020); and increase the risk of diabetes (Huang et al., 2018). According to Benjafield AV et al., China has the largest number of OSA patients, followed by the United States, Brazil, and India; globally, 936 million adults aged between 30 and 69 have OSA, of which 425 million have moderate or severe OSA (Benjafield et al., 2019). Therefore, it is very important to diagnose and treat OSA in a timely and effective manner so as to minimize its negative effects on health and improve quality of life to the maximum extent.

The diagnosis of OSA depends on the matching of clinical manifestations and the objective results of sleep monitoring. The main clinical manifestations are snoring during sleep, daytime drowsiness, fatigue, increased nocturia, and headache. The gold standard for diagnosis is in-lab polysomnography (PSG), but it requires sleep laboratories and trained technicians to monitor sleep throughout the night, making it expensive, technically demanding and time-consuming, so it cannot be widely used for a large number of patients who need to be tested for suspected OSA, especially in hospitals in small and medium-sized cities (Lévy et al., 2015). As such, several simple, effective and easy-to-use screening scales have been developed to identify individuals at risk of OSA, such as the NoSAS score, (Marti-Soler et al., 2016; Hong et al., 2018) STOP-Bang questionnaire, (Chung et al., 2016) GOAL questionnaire, (Duarte R. L. et al., 2020) Berlin questionnaire (Tan et al., 2017), and Epworth Sleepiness Scale (ESS) score (Johns, 1991). GOAL is a recently developed tool for screening OSA hypopnea syndrome. Its sensitivity and specificity in screening OSA in the Brazilian population are similar to those of other screening scales, but no studies have shown that GOAL has been similarly verified in screening OSA in the Asian population. Therefore, in this study, the data of GOAL, STOP-Bang, NoSAS, Berlin, STOP, and ESS is collected and statistically analyzed in order to verify the screening value of GOAL for OSA and compare its predictive ability with those of the other five OSA screening scales.

## Materials and methods

### Study population

The data of 2,171 outpatients and inpatients who underwent PSG (Alice 5, Philips, Amsterdam, USA) examination at the Sleep Medicine Center of the First Affiliated Hospital of Guangzhou Medical University from January 2013 to November 2016 was collected. All patients voluntarily participated in this study and signed informed consent forms. Inclusion criteria: (1) patients who first went to the center to receive PSG monitoring because of sleep breathing disorders; (2) patients aged between 18 and 80 years old; (3) patients who completed the NoSAS score, Epworth Sleepiness Scale, GOAL, the Berlin questionnaire, STOP and STOP-Bang questionnaire in the sleep laboratory; and (4) patients whose total sleep time was more than 4 h. Exclusion criteria: (1) patients with a history of brain tumor or epilepsy; (2) patients treated with sedative or hypnotic drugs for various psychiatric diseases; (3) patients with severe organ failure; (4) OSA patients who received treatment; (5) patients who did not fully complete the scales; and (6) patients with sleep apnea hypopnea syndrome dominated by central or mixed events. This study was approved by the Medical Ethics Committee of the First Affiliated Hospital of Guangzhou Medical University.

### Questionnaire

Before receiving PSG monitoring, the subjects were assessed as follows: (1) GOAL (Duarte R. L. et al., 2020) includes four questions: male, body mass index (BMI) ≥ 30 kg/m^2^, age ≥ 50 years old and loud snoring, answered with “yes” or “no”; “yes” is 1, “no” is 0 and a total score of ≥ 2 indicates a patient at high risk of OSA. (2) NoSAS (Marti-Soler et al., 2016; Hong et al., 2018) includes five questions: neck circumference, BMI, snoring history, age and gender. The total score is 17, of which neck circumference > 40 cm is 4, 25 < BMI < 30 is 3, BMI ≥ 30 is 5, snoring is 2, age ≥ 55 years old is 4 and male is 2, and a score of ≥ 8 indicates a patient at high risk of OSA. (3) STOP-Bang (Chung et al., 2016) includes eight questions: snoring, tiredness, observed apnea, hypertension, BMI > 35 kg/m^2^, age > 50 years old, neck circumference > 40 cm and male, answered with “yes” or “no”; “yes” is 1, “no” is 0 and a total score of ≥ 3 is positive, indicating a patient at high risk of OSA. (4) STOP (Chiu et al., 2017) includes four questions: snoring, tiredness, observed apnea and hypertension, answered with “yes” or “no”; “yes” is 1, “no” is 0 and a total score of ≥ 2 indicates a patient at high risk of OSA. (5) Berlin (Tan et al., 2017) includes 11 questions in three groups: (1) severity of snoring; (2) drowsiness within 2 days; (3) hypertension or obesity. After calculating the score, each group is evaluated as negative or positive. If the positive value of the three groups is greater than or equal to that of the two groups, it indicates a patient at high risk of OSA. (6) ESS (Johns, 1991) includes eight questions: subjects are asked to assess their degree of dozing off in specific scenarios during the day, with 0 as no dozing and 1, 2, and 3 as mild, moderate and severe dozing respectively. The total score is 24, and a score of ≥ 9 is positive. At the beginning, the main purpose of establishing ESS was to evaluate daytime sleepiness, but in recent years, it has been explored as a potential OSA screening tool.

Apnea Hypopnea Index (AHI) refers to the number of apneas or hypopneas per hour of sleep. The diagnosis of OSA was based on the third edition of the *International Classification of Sleep Disorders* issued by the American Sleep Medical Association, and was graded according to AHI: normal group (AHI < 5 events/h), mild OSA group (5 ≤ AHI < 15 events/h), moderate OSA group (15 ≤ AHI < 30 events/h), and severe OSA group (≥ 30 events/h) (Berry et al., 2012).

### Statistical processing

SPSS23.0 statistical software was used for analysis. Continuous variables were expressed as mean ± standard deviation or median and interquartile range according to variable distribution. and the categorical data was expressed by frequency. Continuous variables were tested by single factor analysis of variance, and the categorical data was tested by the *X*^2^ test. The subjects’ working characteristic (ROC) curves were analyzed and evaluated by MedCalc 11.5.1 (MedCalc Software, Ostend, Belgium) software. The sensitivity, specificity, positive predictive value and negative predictive value of the five scales were calculated in the form of a four-grid table and reported with a 95% confidence interval (CI). The diagnostic value for correctly identifying individuals with OSA of GOAL and the other five screening scales was evaluated by comprehensively comparing the area under the curve, sensitivity and specificity of each scale. P < 0.05 was defined as statistically significant.

## Results

### Basic data

Among the 2,171 subjects included in this study, 1,696 were male, accounting for 78.1%, and the gender difference was statistically significant (*P* < 0.001) (see Table 1 for details). Among the 2,171 subjects, their average scores for GOAL, NoSAS, STOP-Bang, STOP, ESS, and Berlin were 2.2 ± 1.0, 8.6 ± 3.9, 3.5 ± 1.5, 1.9 ± 1.0, 7.9 ± 5.7, and 1.5 ± 0.9. There were 1,507 OSA patients, accounting for about 69.4%. Among them, there were 458 patients (21.1%) with mild OSA, 349 (16.1%) with moderate OSA and 700 (32.2%) with severe OSA. Moderate to severe OSA patients accounted for 48.3%. The lowest nocturnal oxygen saturation was 77.6 ± 14.9%. The average AHI, neck circumference and waist circumference were 24.5 ± 25.6 events/h, 38.4 ± 3.9 cm and 95.3 ± 13.5 cm respectively. The lowest nocturnal oxygen saturation, neck circumference, AHI and waist circumference increased with the severity of OSA. In this study, there was no significant difference in age between the normal group and severe OSA group, and there was no significant difference in average nocturnal oxygen saturation between the normal group and mild OSA group.

**TABLE 1:** Basic data of patients in each group and scores of six scales.

Project	All	Normal group	Mild OSA	Moderate OSA	Severe OSA	*P*-value
Number of cases	2,171	664	458	349	700	-
GOAL^b^	2.2 ± 1.0	1.4 ± 1.0	2.4 ± 0.7	2.5 ± 0.7	2.7 ± 0.8	< 0.001
NoSAS^b^	8.6 ± 3.9	6.5 ± 3.8	8.4 ± 3.4	9.2 ± 3.3	10.5 ± 3.3	< 0.001
STOP-Bang^b^	3.5 ± 1.5	2.7 ± 1.3	3.4 ± 1.3	3.8 ± 1.4	4.2 ± 1.4	< 0.001
STOP^b^	1.9 ± 1.0	1.4 ± 0.9	1.8 ± 0.9	2.0 ± 1.0	2.3 ± 1.1	< 0.001
ESS^b^	7.9 ± 5.7	6.2 ± 5.1	7.2 ± 5.2	7.3 ± 5.2	10.2 ± 6.0	< 0.001
Berlin^b^	1.5 ± 0.9	1.0 ± 0.8	1.5 ± 0.8	1.7 ± 0.8	2.0 ± 0.8	< 0.001
AHI (events/h)^b^	24.5 ± 25.6	1.87 ± 1.4	9.4 ± 2.7	21.3 ± 4.1	57.4 ± 17.3	< 0.001
BMI (kg/m^2^)^b^	26.5 ± 4.1	24.8 ± 4.0	26 ± 3.5	26.6 ± 3.7	28.3 ± 4.1	< 0.001
Waistline (cm)^b^	95.3 ± 13.5	89.7 ± 11	93.7 ± 10.9	96 ± 10.2	101.2 ± 16	< 0.001
Age (years)^b^	47.6 ± 13.9	47.1 ± 14.7	49.7 ± 13.1	49.8 ± 14.1	45.6 ± 13.1	< 0.001
Average night blood oxygen saturation (%)^b^	94.2 ± 3.6	95.7 ± 2.2	95.5 ± 2.0	95.0 ± 2.1	91.6 ± 4.6	< 0.001
Minimum night blood oxygen saturation (%)^b^	78.1 ± 13.8	88.2 ± 6.1	82.3 ± 9.2	78.0 ± 8.7	65.8 ± 14.2	< 0.001
Neck circumference (cm)^b^	38.4 ± 3.9	36.4 ± 3.9	37.9 ± 3.6	38.6 ± 3.3	40.4 ± 3.4	< 0.001
Male (%)^a^	1,696 (78.1)	436 (65.7)	342 (74.6)	279 (79.9)	639 (91.3)	< 0.001

^a^frequency and chi-square test; ^b^mean and variance, and one-way analysis of variance.

### Predictive value of six scales

**FIGURE 1:**
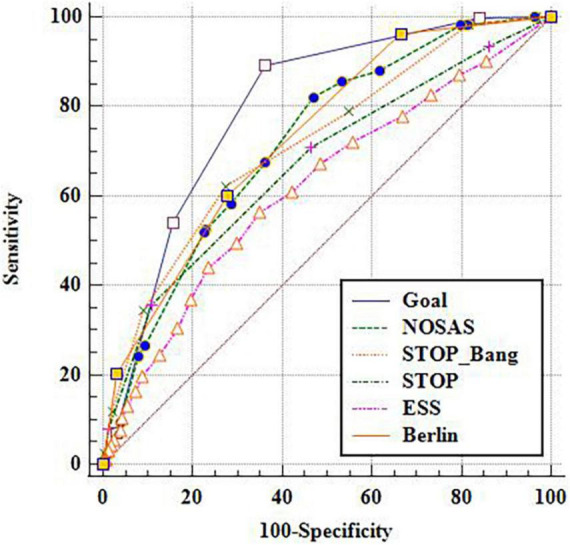
ROC curve with apnea hypopnea index (AHI) 5 as cut-off point.

**FIGURE 2:**
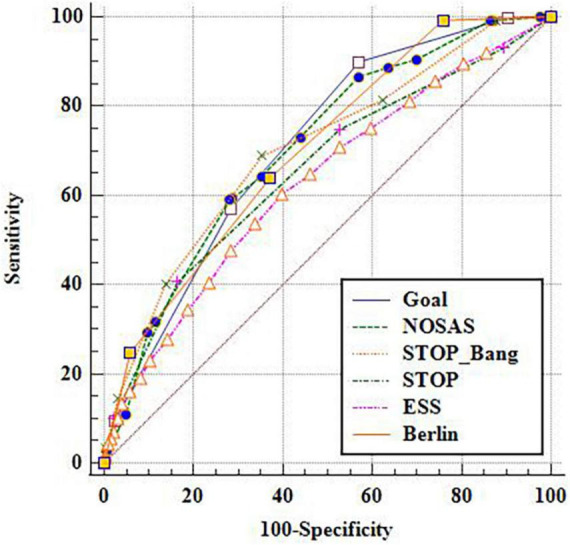
ROC curve with apnea hypopnea index (AHI) 15 as cut-off point.

**FIGURE 3:**
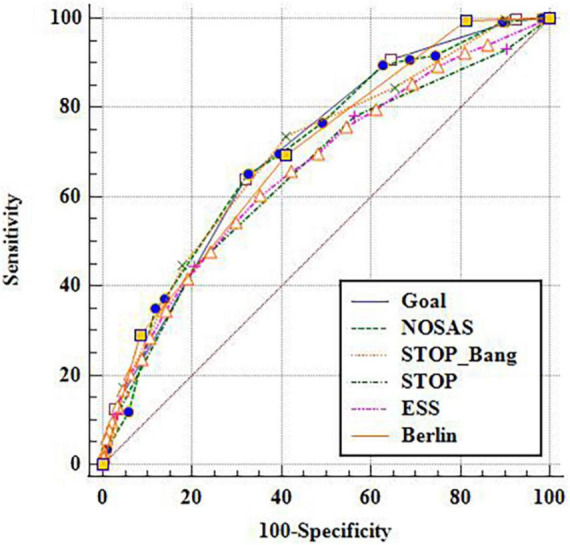
ROC curve with apnea hypopnea index (AHI) 30 as cut-off point.

Taking the cut-off point as AHI of 5, 15 and 30 events/h respectively, the ROC under the curve (AUC) of GOAL and the other five scales were compared (Figures 1–3). It is found that GOAL questionnaire had comparable screening ability to the NoSAS and STOP-BANG, and performed better than the ESS (the AUC scores of STOP and ESS were less than 70%). When the cut-off point of AHI was 5 events/h, the AUC of GOAL was the highest at 0.799 (0.781–0.816). The AUC scores of NoSAS, STOP-Bang and Berlin were 0.720 (0.701–0.739), 0.719 (0.700–0.738), and 0.734 (0.715–0.752) respectively (see Figures 1–3).

### Prediction indexes of six scales

With the aggravation of OSA, the sensitivity and negative predictive value of the six screening scales increased, while their specificity and positive predictive value decreased. The sensitivity, negative predictive value, specificity, and positive predictive value of GOAL ranged from 0.891 to 0.905, 0.716 to 0.886, 0.422 to 0.349, and 0.843 to 0.398 respectively in three cut-off points. When the cut-off point of AHI was 5 events/h, the sensitivity of GOAL was the highest, while ESS and Berlin had higher specificity but lower sensitivity (see Tables 2–4).

**TABLE 2:** Taking apnea hypopnea index (AHI) 5 as cut-off point for diagnosis of obstructive sleep apnea (OSA).

Questionnaire	ROC	Sensitivity	Specificity	PPV	NPV
GOAL	0.799 (0.781∼0.816)	0.891 (0.875∼0.906)	0.625 (0.588∼0.661)	0.843 (0.826∼0.861)	0.716 (0.679∼0.752)
NoSAS	0.720 (0.701∼0.739)	0.674 (0.651∼0.699)	0.637 (0.600∼0.673)	0.808 (0.787∼0.830)	0.463 (0.431∼0.496)
STOP-Bang	0.719 (0.700∼0.738)	0.788 (0.767∼0.808)	0.452 (0.414∼0.490)	0.765 (0.744∼0.786)	0.484 (0.445∼0.523)
STOP	0.671 (0.651∼0.691)	0.708 (0.685∼0.731)	0.536 (0.498∼0.574)	0.776 (0.754∼0.798)	0.447 (0.413∼0.482)
ESS	0.622 (0.601∼0.643)	0.493 (0.468∼0.518)	0.702 (0.667∼0.737)	0.790 (0.764∼0.816)	0.379 (0.352∼0.406)
Berlin	0.734 (0.715∼0.752)	0.600 (0.575∼0.625)	0.723 (0.689∼0.757)	0.831 (0.809∼0.853)	0.443 (0.414∼0.473)

**TABLE 3:** Taking apnea hypopnea index (AHI) 15 as cut-off point for diagnosis of obstructive sleep apnea (OSA).

Questionnaire	ROC	Sensitivity	Specificity	PPV	NPV
GOAL	0.711 (0.691∼0.730)	0.898 (0.880∼0.916)	0.422 (0.393∼0.450)	0.592 (0.568∼0.616)	0.816 (0.784∼0.847)
NoSAS	0.707 (0.688∼0.726)	0.730 (0.703∼0.757)	0.561 (0.532∼0.591)	0.609 (0.582∼0.636)	0.690 (0.660∼0.720)
STOP-Bang	0.704 (0.684∼0.723)	0.813 (0.790∼0.837)	0.378 (0.350∼0.406)	0.550 (0.525∼0.574)	0.684 (0.647∼0.720)
STOP	0.656 (0.636∼0.676)	0.844 (0.817∼0.871)	0.347 (0.323∼0.372)	0.381 (0.357∼0.405)	0.824 (0.794∼0.854)
ESS	0.629 (0.609∼0.650)	0.535 (0.505∼0.565)	0.661 (0.634∼0.689)	0.596 (0.565∼0.628)	0.603 (0.576∼0.631)
Berlin	0.703 (0.683∼0.722)	0.640 (0.611∼0.669)	0.628 (0.600∼0.657)	0.617 (0.588∼0.646)	0.651 (0.623∼0.679)

**TABLE 4:** Taking apnea hypopnea index (AHI) 30 as cut-off point for diagnosis of obstructive sleep apnea (OSA).

Questionnaire	ROC	Sensitivity	Specificity	PPV	NPV
GOAL	0.708 (0.688∼0.727)	0.905 (0.884∼0.927)	0.349 (0.325∼0.374)	0.398 (0.374∼0.423)	0.886 (0.860∼0.912)
NoSAS	0.706 (0.686∼0.725)	0.766 (0.734∼0.797)	0.509 (0.484∼0.535)	0.426 (0.399∼0.453)	0.820 (0.795∼0.845)
STOP-Bang	0.701 (0.681∼0.720)	0.844 (0.817∼0.871)	0.347 (0.323∼0.372)	0.381 (0.357∼0.405)	0.824 (0.794∼0.854)
STOP	0.653 (0.632∼0.673)	0.780 (0.749∼0.810)	0.436 (0.411∼0.462)	0.397 (0.371∼0.423)	0.807 (0.779∼0.834)
ESS	0.666 (0.645∼0.685)	0.601 (0.565∼0.638)	0.647 (0.622∼0.671)	0.447 (0.416∼0.479)	0.773 (0.750∼0.797)
Berlin	0.699 (0.679∼0.718)	0.693 (0.659∼0.727)	0.590 (0.565∼0.615)	0.446 (0.416∼0.475)	0.801 (0.777∼0.825)

## Discussion

In our study, there were 1,507 OSA patients among 2,171 subjects, accounting for about 69.4%. This high prevalence is because the patients who came for PSG monitoring were mainly patients with suspected OSA. Among them, there were 458 patients (21.1%) with mild OSA, 349 (16.1%) with moderate OSA and 700 (32.2%) with severe OSA. Regardless of the cut-off point, the AUC score reveals that GOAL questionnaire had comparable screening ability to the NoSAS and STOP-BANG, and performed better than the ESS. Taking AHI ≥ 5 events/h as the diagnostic standard, the sensitivity of GOAL was the highest, while ESS and Berlin had higher specificity but lower sensitivity. These findings suggest that GOAL has high screening value for OSA. OSA is a respiratory sleep disorder characterized by recurrent episodes of the partial or complete obstruction of the upper respiratory tract at night, resulting in intermittent hypoxia and hypercapnia (Maniaci et al., 2021). Metabolic syndrome is a group of potential risk factors for cardiovascular and metabolic diseases, including abdominal obesity, dyslipidemia, hypertension, insulin resistance, elevated blood sugar and so on (Borel, 2019). Several studies have reported that OSA is closely related to metabolic syndrome, obesity, BMI, large waistline and cardiovascular disease (Mazzuca et al., 2014; Borel, 2019; Yeghiazarians et al., 2021). In this study, the proportion of males was much higher than that of females, and the neck circumference and waistline of OSA patients were higher than those of the subjects in the normal group. This is consistent with the current research results.

STOP-Bang was originally developed for OSA screening in patients undergoing preoperative surgery. It has been reported that STOP-Bang is more suitable for OSA screening than Berlin, STOP or ESS (Chiu et al., 2017). NoSAS is a new tool that was developed in a Swiss cohort and subsequently verified by a Brazilian team, and studies have shown that it demonstrates higher screening ability than Berlin and STOP-Bang (Herschmann et al., 2021). Berlin was developed by a group of respiratory and primary care doctors to screen for high-risk OSA. It is commonly used in epidemiological and clinical studies, and has variable results in terms of sensitivity and specificity (Ng et al., 2019). An ideal screening tool should have high sensitivity and specificity, and large AUC (Duarte et al., 2021). This study found that no matter which cut-off point was used, the AUC score of GOAL reveals that GOAL questionnaire had comparable screening ability to the NoSAS and STOP-BANG, and performed better than the ESS. The results of this study show that the screening effects of GOAL, NoSAS, and STOP-Bang in the Chinese population are better, while those of STOP and ESS are inferior.

Duarte RLM et al. pointed out that no matter which cohort, with the increase in the severity of OSA, sensitivity of GOAL questionnaire increased up to 94.5% and specificity decreased (Duarte et al., 2021). GOAL had higher sensitivity than STOP-Bang and NoSAS in screening OSA. The sensitivity of GOAL was the higher, and the scores of STOP-Bang and NoSAS also had higher sensitivity. When AHI ≥ 30 events/h, the sensitivity of GOAL was the highest, reaching 90.5%. The lowest score of GOAL was 90.5%. The more serious the disease, the higher the sensitivity of ESS and the lower its specificity, which is consistent with the current study (Duarte R. L. M. et al., 2020). The Youden index is the sum of sensitivity and specificity minus 1. The larger the index, the better the effect of the screening experiment and the greater the authenticity. Obviously, the Youden index of GOAL questionnaire is not different from NoSAS and STOP-Bang. For patients suspected of OSA coming to the hospital for examination, it is better not to miss diagnosis, so a highly sensitive screening questionnaire is required. From this perspective, GOAL questionnaire, the NoSAS and STOP-BANG questionnaire are more appropriate. Although the specificity of GOAL questionaire is low, the sensitivity of GOAL questionnaire is high, so GOAL questionnaire’s Youden index is not lower than other questionnaires, which also indicates that GOAL questionaire has good diagnostic efficacy.

GOAL consists of four items which are easily available and recognized as predictors of OSA: gender, BMI, age, and snoring. It has fewer items compared with ESS. Compared with NoSAS and STOP-Bang, which are currently more effective in screening OSA, they have similar AUC, sensitivity, specificity, positive predictive value and negative predictive value, with some even better. GOAL lacks only the neck circumference item compared with NoSAS and is easier to implement, so GOAL is more sensitive, but its specificity is lower than that of NoSAS. From this study, we can see that both GOAL and NoSAS can be used as simple questionnaire tools for screening OSA, which is worth popularizing, especially in areas where resources are scarce and sleep apnea detection equipment such as PSG is not available (Roche et al., 2021; Zeng et al., 2021). Early diagnosis and treatment is very important for the prognosis of OSA patients because it can reduce various cardio-cerebrovascular complications and medical burdens caused by long-term nocturnal hypoxia-induced oxidative stress (Zheng et al., 2021).

### Limitations of this study

As a retrospective study of patients from a single center, this study is mainly based on the population of Guangdong Province and cannot represent the broad population of China. However, as the National Respiratory Medicine Center, its patients come from all over the country, which can somewhat make up for the above deficiency. The contents of the questionnaires used in this study should be recorded in accordance with a strict process before a sleep test is carried out in our sleep center, but the questionnaires were completed by the patients with their families. In the future, a national multi-center large-scale study and Shenzhen-based international multi-center joint research should be further promoted to explore the clinical effectiveness of these screening tools.

To sum up, in the screening of OSA, GOAL is a free, efficient and easy to manage tool with a screening ability not lower than those of NoSAS and STOP-Bang, and better than that of ESS.

## Data availability statement

The raw data supporting the conclusions of this article will be made available by the authors, without undue reservation.

## Ethics statement

The studies involving human participants were reviewed and approved by Medical Ethics Committee of the First Affiliated Hospital of Guangzhou Medical University. Written informed consent for participation was not required for this study in accordance with the national legislation and the institutional requirements. Written informed consent was obtained from the individual(s) for the publication of any potentially identifiable images or data included in this article.

## Author contributions

WL, JZ, HL, and MC were the guarantor of the manuscript and took responsibility for the content of this manuscript. ZZ, MC, RC, and CW contributed to the design and data analysis. CL, HL, and RC contributed to the acquisition of primary data. ZZ, JZ, and HL wrote the initial draft of the manuscript. KW, RC, and WL contributed significantly to the revision of the manuscript. All authors read and approved the final manuscript.
